# Monocular perceptual learning of contrast detection facilitates binocular combination in adults with anisometropic amblyopia

**DOI:** 10.1038/srep20187

**Published:** 2016-02-01

**Authors:** Zidong Chen, Jinrong Li, Jing Liu, Xiaoxiao Cai, Junpeng Yuan, Daming Deng, Minbin Yu

**Affiliations:** 1 State Key Laboratory of Ophthalmology, Zhongshan Ophthalmic Center, Sun Yat-sen University, Guangzhou, China

## Abstract

Perceptual learning in contrast detection improves monocular visual function in adults with anisometropic amblyopia; however, its effect on binocular combination remains unknown. Given that the amblyopic visual system suffers from pronounced binocular functional loss, it is important to address how the amblyopic visual system responds to such training strategies under binocular viewing conditions. Anisometropic amblyopes (n = 13) were asked to complete two psychophysical supra-threshold binocular summation tasks: (1) binocular phase combination and (2) dichoptic global motion coherence before and after monocular training to investigate this question. We showed that these participants benefited from monocular training in terms of binocular combination. More importantly, the improvements observed with the area under log CSF (AULCSF) were found to be correlated with the improvements in binocular phase combination.

Amblyopia is a developmental disorder affecting both monocular and binocular functions following abnormal early visual experience^1^. Unequal refractive error (anisometropia), an eye turn (strabismus) and lack of visual inputs (deprivation) are common causes of amblyopia^2^. Traditionally, it is believed that amblyopia is no longer treatable after its critical period of visual plasticity^3^,^4^. A new era is on the horizon, whereby we have witnessed adults with amblyopia can still be treated after interventional treatment^5^,^6^,^7^,^8^,^9^,^10^. Perceptual learning is one of the most promising methods available nowadays with emerging evidence showing improvement in both monocular and binocular functions as observed after intensive training^11^,^12^,^13^,^14^,^15^.

The mechanism of how perceptual learning affects both monocular as well as binocular visual functions remains to be answered. Hess and colleagues have investigated abnormal binocular interaction as the main cause of amblyopic syndromes [reduced visual acuities (VA), stereopsis, *et al.*^15^,^16^,^17^,^18^,^19^] and have developed a novel anti-suppression therapy to counteract this imbalance^20^,^21^,^22^. However, there is also evidence that monocular treatment can still result in better stereo-acuity and promote binocular fusion in some cases^23^,^24^. This implies that monocular treatment has certain rehabilitative effects on binocular functions. Recent studies also argued that VA improvement does not necessarily correlate with interocular suppression elimination^25^. It is possible because task promoting monocular function is embodied in dichoptic perceptual learning, which might result in enhanced binocular function due to mixed mechanism (both monocular and binocular). We realized that in our previous study^15^, the monocular training group only employed a monocular falling blocks game where its training effects might not have been optimized. Meanwhile, the contrast sensitivity function (CSF), which reflects an overall spatial frequency tuning response of V1 neurons^26^,^27^, is a more sensitive measurement to monitor monocular performance^28^,^29^,^30^ as compared to VA alone. Therefore, there is a need to clarify whether or not monocular training facilitate binocular combination in amblyopia, and what its mechanism involves using more appropriate parameters.

Binocular function in amblyopia is measured clinically using the Worth-4-dot test and the Bagolini striated lens. These qualitative measurements; however, provide gross information about binocular combination abilities. While stereo-acuity is widely used, it is strongly dependent on VAs and not ideal for monitoring detailed changes of rudimentary binocular functions. Recently, several quantitative methods have been introduced and successfully applied in amblyopic patients. For example, a method under the framework of global motion coherence is available to quantify interocular suppression^31^,^32^,^33^ and has been successfully applied in the clinical population^19^,^34^,^35^,^36^. Meanwhile, Huang and colleagues have developed a binocular cyclopean phase combination and contrast matching task^37^,^38^ based on the contrast gain-control theory introduced by Ding^39^,^40^. This method has been proven to be a rapid and robust clinical measurement, even for young observers^41^. More recently, the regional distribution of interocular suppression among different types of amblyopes has been explored, shedding some light on their suppressive features^42^.

In this study, we chose a well-accepted monocular perceptual learning paradigm to treat anisometropic amblyopes aged beyond their critical period. We made direct comparisons of binocular functions before and after treatment using two newly developed quantitative methods (Fig. 1B,C). By using the area under curve log CSF (AULCSF) (Fig. 1A) as an indicator of monocular function, we found that monocular function improvement in the amblyopic eyes indeed affected binocular combination in amblyopia.

## Methods

### Participants

Only unilateral anisometropic amblyopes were recruited in this study. All refractive corrections were assessed by cycloplegic refraction. Both anterior (by slit lamp examination) and posterior (by direct ophthalmoscopy) segments were examined to exclude organic causes during the first visit. Unilateral anisometropic amblyopes (n = 13) were recruited and assigned to the perceptual learning (PL) group. Another 10 participants with matched degrees of anisometropic amblyopia (as indicated by VA of the amblyopic eye) were recruited for the test-retest reliability test. Detail information of baseline characteristics is provided in Table 1 (see also in Table S1). Written informed consent was obtained from each participant. The research adhered to the tenets of the Declaration of Helsinki and the experimental protocol was reviewed and approved by The Zhongshan Ophthalmic Center Ethics Committee. Amblyopia was defined as an interocular VA difference of at least 0.2 logMAR with no organic cause. Anisometropia was defined as a spherical equivalent difference of 1.50 diopter or more between the two eyes. All participants were naïve observers to psychophysical tasks.

### Visual acuity and stereo-acuity

Visual acuity was measured using the Tumbling E-ETDRS chart and expressed in logMAR units. Best corrected VAs were assessed before, during and after training for the PL group. For the participants who received patching, their VAs were measured before and after treatment only. Stereo-acuity was assessed using the Randot Preschool Test whereby stereo-sensitivity was defined as the reciprocal of stereo-acuity (1/arcmin).

### Training

Participants were trained within one log-unit from their cut-off spatial frequencies (details can be found in SI). The training paradigm was an observation task similar to that used in CSF assessment. Training began from the day CSF were determined and lasted for seven to twelve days. Each participant performed ten training sessions a day and each session consisted of 70 ~ 100 trials. Overall, each participant received about 5,000 ~ 10,000 trials, totaling around eight hours of training before an endpoint measurement was made.

### Patching period of the participants for the test-retest reliability check

Participants for the test-retest reliability were recruited occasionally from the clinic and thus were not well matched with the PL group in terms of age and follow-up period. Though any intervention might confound the result, patching was instructed in order to adhere to the clinical routine because of age of these participants (most of them at the age around 10 years). Their non-amblyopic eyes were patched with full correction for 2–4 hours/day. Details could be found in SI. This intervention would inevitably affect the interpretation of the results and will be discussed later.

## Psychophysics measurements

### Contrast sensitivity function (CSF)

The stimuli were first order sinusoidal gratings generated by a psychophysical software Psykinematix^43^ installed in a MacBook Pro laptop presented on a gamma-calibrated Dell 17-inch color CRT monitor (refresh rate = 85 Hz), at a 10.8 bits monochromatic mode to ensure high grayscale resolution. Mean luminance was 50cd/m^2^. Stimuli were viewed monocularly under dim illumination at a 120 cm working distance on a movable chinrest in order to project a 2 degree angular subtense by the sinusoidal stimuli.

Contrast sensitivity was defined as the reciprocal of contrast threshold. The stimulus edge was blurred by a half-Gaussian ramp of 0.5 degrees. Each stimulus was oriented either horizontally or vertically presented at an interval of 120 ms and participants were asked to judge its orientation. A two-alternative forced choice (2AFC) method with an adaptive three-down one-up staircase was applied to determine the contrast threshold. Contrast threshold was defined as an average of the last four reversals of the staircase. In this case, thresholds at 79.3% correctness were calculated. The CSF was obtained in the non-amblyopic and amblyopic eyes across spatial frequencies of 0.5, 1, 2, 4, 8, 12, 16 cycles per degree (cpd). Cut-off was defined as the spatial frequency at which contrast threshold was higher than 0.5^12^. Testing order of eyes and spatial frequencies were randomized. Each participant observed about 500 trials at low spatial frequencies to familiarize themselves with the procedure prior to data collection.

Three normal participants (details are given in SI), additional test was made with different densities of ND filters (Kodak Wratten; Eastman Kodak, Rochester, NY; ND filter bar; ND filters were piled to give 0.3-log unit increments). CSFs of the three normal participants were measured under the same condition but with a non-activated shutter goggle.

### Quantitative measurements of binocular function

Dichoptic stimuli to measure binocular functions were presented on an OLED goggle (Z800 Pro, eMagin Corp., Washington, DC). The stimuli were generated by a laptop (MacBook Pro) using the Matlab PsychToolBox 3.0.9 extension (version 2012)^44^,^45^. The screen displayed a 40 degree angle, with a resolution of 800*600 in each eye. The refresh rate was 60 Hz with a background luminance of 150 cd/m^2^.

Participants PL 2, PL 3, PL 5, PL 7 to PL 13 (n = 10) also observed a binocular phase combination task. The phase combination stimulus was presented on a 27-inch LED monitor (ASUS) with an active shutter stereo-goggle (NVIDIA 3D Vision 2) at the mean luminance of 150 cd/m^2^. The monitor was gamma calibrated at refresh rate of 120 Hz to ensure 60 Hz presentation in each eye. Stimulus parameters were carefully adjusted to be consistent with the visual angle of the OLED goggles. Noted that as the shutter goggles halved the mean luminance, comparison was only made between measurements using the same device.

### Binocular phase combination

The binocular phase combination task was similar to previously described^33^,^37^. Two horizontal sinusoidal gratings of identical spatial frequencies and orientation with a 45^o^ offset phase difference were presented dichopticly. The grating contrast was fixed at 100% in the amblyopic eye. Two grating configurations (with either +22.5^o^ or −22.5^o^ of phase) were used to cancel the potential bias of upward or downward preference. The resultant phase shift was defined as the subtraction of these two configurations and used to calculate the effective contrast ratio in this task. Adjustment was made at a step size of 4^o^. The gratings contained two complete cycles at a spatial frequency of 0.293 cpd. The program measured phase difference with interocular contrast ratios at 0, 0.1, 0.2, 0.4, 0.8 and 1.0. Each pair of interocular contrast ratio was repeated in four blocks.

### Dichoptic global motion coherence

The idea of dichoptic global motion coherence was to manipulate interocular contrast ratio in order to form binocular perception of both the signal and noise percept. Details can be found elsewhere^34^. In brief, we presented signal as well as noise dots to both eyes to obtain a motion coherence ratio, which was assumed to be constant through measurements. The signal and noise were then separated and presented to the amblyopic and non-amblyopic eyes respectively at their fixed motion coherence ratio. An adaptive three-down, one-up staircase was applied to control the contrast of the noise dots while the contrast of signal dots was fixed at 100%. The contrast threshold represented the effective contrast in the amblyopic eye to form binocular motion perception. The ratio between the contrast of fellow/amblyopic eye was defined as effective contrast ratio (ECR). All the dots moved at a speed of 2^o^/s, and a maximum of 50 dots were presented in each frame. Dot size was randomized in a range of ±20% around the mean of 1.1^o^. Any dot had a 30% chance to be redrawn in a random position from one frame to the next.

### Modeling and fitting

#### Area under log CSF (AULCSF)

CSF was fitted by a parabolic function in a log-log scale (base 10 for CS and 2 for spatial frequency). Data-points lower than 0.17 (contrast threshold higher than 0.65) were excluded from the fit. The goodness of fit was all above 95%. The AULCSF was determined by calculating the definite integration of the best-fitted function from 0.5 cpd to the root.

### Improvement after training

Both VA and AULCSF improvements were defined as: (Measurement_post_ – Measurement_pre_)/ Measurement_pre_.

### Bandwidth of perceptual learning

A perceptual learning bandwidth was estimated similar to the methods introduced by Huang^12^. Briefly, contrast sensitivity improvement for each trained spatial frequency was normalized to the trained spatial frequency. Spatial frequencies were also normalized based on their distance from trained spatial frequency. Group averaged improvement was fitted with a Gaussian function:


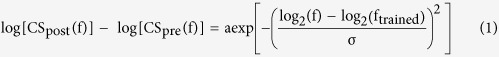


The bandwidth of perceptual learning is defined as: 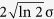.

### Binocular contrast gain control model of phase combination

Data fit was plotted with the Curve Fitting toolbox incorporated in Matlab (version 2012) using a nonlinear least squares method. The data from binocular phase combination task were fitted using a modified gain-control model^37^:


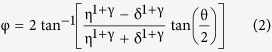


In this model, φ represented the measured phase shift between two configurations where θ was fixed at 45^o^. δ and γ represented the interocular contrast ratio and transducer non-linearity respectively. Therefore, we obtained the 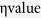, which represented the effective contrast ratio (ECR) of the amblyopic eye from the best-fitted function.

## Results

### Perceptual learning leads to improvements in monocular function

#### CSF and VA at baseline

**Figure 2:**
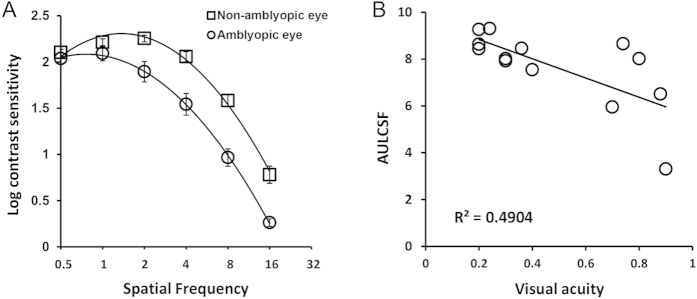
CSFs and VA for the PL group. (**A**) Best fitting curves of CSFs in the amblyopic and non-amblyopic eyes; (**B**) the relationship between AULCSF and VA in the amblyopic eyes. Error bars stand for ±S.E.M.

Figure 2 shows the baseline interocular difference in contrast sensitivities among participants before training. Within subject ANOVA shows that the non-amblyopic eye manifested significantly higher contrast sensitivity than their amblyopic counterpart (F_1,25_ = 43.06, p < 0.0001) and this difference is spatial frequency dependent (F_3,75_ = 9.28, p < 0.0001). The AULCSF correlates with the depth of amblyopia as determined by VA of the amblyopic eye (Pearson’s r = 0.693, p = 0.009).

### Learning curves

**Figure 3:**
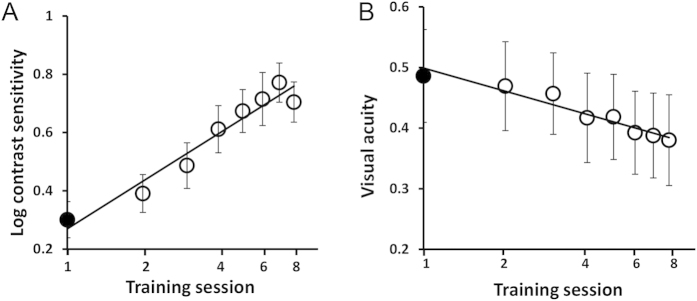
Learning curves of (**A**) contrast sensitivity at the trained spatial frequency and (**B**) visual acuity as functions of training sessions. Only data obtained from the common first eight sessions are shown. Pre-training measurements are labeled in black. Error bars stand for ±S.E.M.

Figure 3 shows that contrast detection training near their cut-off spatial frequency significantly improves contrast sensitivity in a session dependent manner (F_1,7_ = 31.16, p < 0.0001). At the trained spatial frequency, a 3.5 ± 2.15 fold (9.7 ± 4.39 dB) improvement of CS from baseline was observed. VA of the amblyopic eye also improves significantly after training from 0.48 ± 0.27 logMAR to 0.31 ± 0.25 logMAR (t = 7.33, p < 0.0001). Learning curves of contrast sensitivity and VA across training sessions are fitted by log-log linear functions with slopes of 0.54 (r^2^ = 0.94, p < 0.0001) and −0.13 (r^2^ = 0.93, p = 0.002) respectively.

### CSFs and VA before and after training

**Figure 4:**
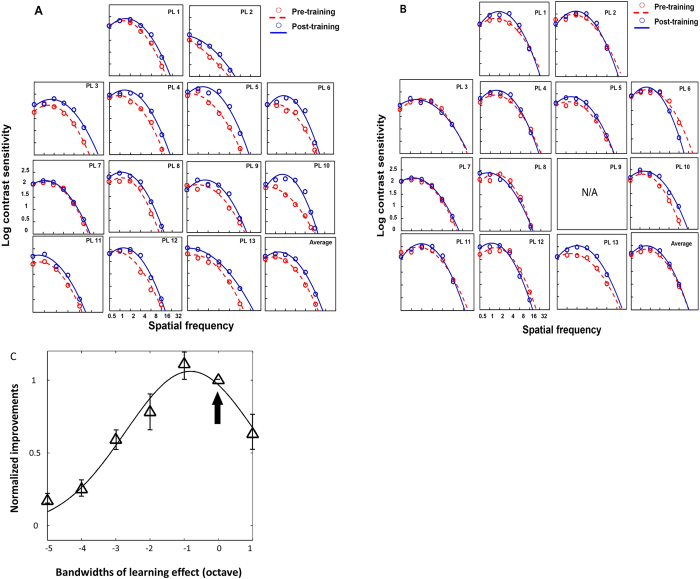
Contrast sensitivity functions before and after perceptual learning in (**A**) the amblyopic eyes; (**B**) the non-amblyopic eyes, group average fitting is show in the last graph. (**C**) Bandwidth of perceptual learning in the amblyopic eye. Only data from participants who trained at a single spatial frequency was analysis. Arrow indicating trained spatial frequency. Error bars stand for ±S.E.M.

The effect of perceptual learning at a specific spatial frequency transferred to the untrained spatial frequencies as well as VA. VA significantly improves by 1.64 ± 0.06 lines (paired t-test: t_12_ = 6.49, p < 0.0001). Within subject ANOVA shows that CSFs of the amblyopic eyes vary before and after training (F_1,25_ = 22.9, p < 0.0001) and it varies with spatial frequency (F_3,75_ = 425.3, p < 0.0001). Interaction of the two factors is significant (F_3,75_ = 5.9, p = 0.03), which means that the CS improvements depend on spatial frequency (Fig. 4A). On the other hand, training of the amblyopic eye doesn’t lead to significant improvement in the untrained non-amblyopic eyes (F_1,25_ = 2.57, p = 0.122) (Fig. 4B).

Figure 4C shows that training effect transferred to untrained spatial frequency with the bandwidth of 3.73 ± 0.53 octaves (r^2^ = 0.97). Our result is consistent with previous reports showing that amblyopia has a broader bandwidth of perceptual learning than normal observers.

### Perceptual learning lead to improvements in binocular function

#### Binocular combination after perceptual learning

**Figure 5:**
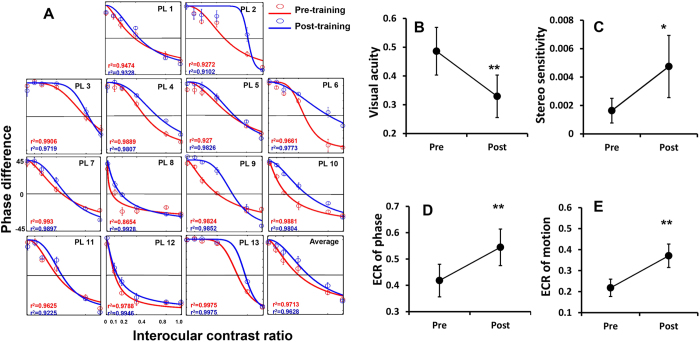
Binocular phase combination before and after treatment: (**A**) individual fits with Huang’s model (2009). Solid lines represent the best fitted curves according to the model. Red and blue lines stand for the measurements before and after treatment, respectively. ECRs are indicated where the curves cross the line of zero. Group average fitting is shown in the last graph. (**B–E**) Summary of visual functions before and after training. (**B**) visual acuity, (**C**) stereo-sensitivity; (**D**) ECR of binocular phase combination and (**E**) ECR of dichoptic motion coherence. The asterisks indicate significance level. *p < 0.05; **p < 0.01.

Individual data of phase combination task are fitted according to the model with two free parameters introduced by Huang^37^ and shown in Fig. 5A. Overall, the model provides good fits to the data with the means of r^2^  = 0.962 and 0.969 before and after training. In summary, monocular perceptual learning does result in certain improvement of binocular function in different tasks (Fig. 5B–E). Stereo sensitivity increases significantly after training (paired t test: t_12_ = 2.35, p = 0.037). The average ECR of phase increases significantly from 0.43 ± 0.21 before training to 0.57 ± 0.22 (paired t test, t_12_ = 4.72, p = 0.0005), while the transducer nonlinearity (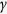) remains largely unchanged (pre: 1.27 ± 1.44; post: 3.20 ± 5.02; paired t test, t_12_ = 1.50, p = 0.119). ECR of dichoptic global motion coherence task also exhibits a similar pattern by increasing from 0.22 ± 0.13 to 0.34 ± 0.17 (paired t test, t_9_ = 4.19, p = 0.002).

### Test-retest reliability

**Figure 6:**
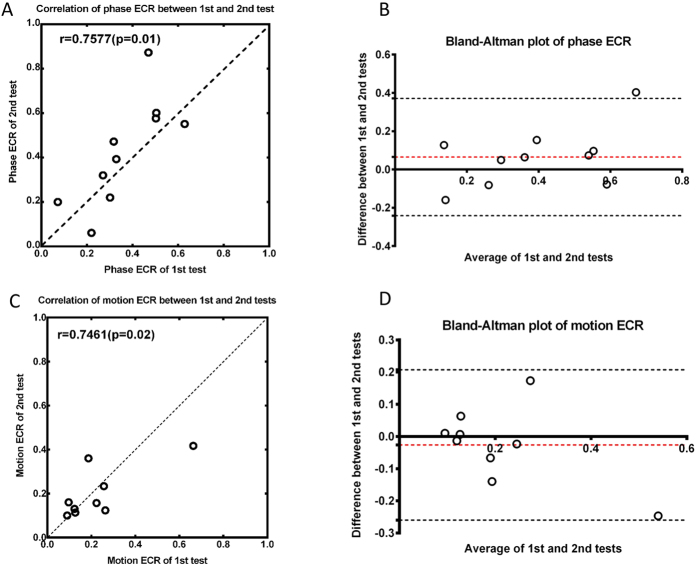
Test-retest reliability of the binocular function measurements. (**A**,**C**) correlation between 1^st^ and 2^nd^ tests of (**A**) binocular phase combination and (**C**) dichoptic motion coherence. The dotted line represents the line of equality. (**B**,**D**) plots of difference of ECR between 1^st^ and 2^nd^ tests against the average of them for (**B**) binocular phase combination and (**D**) dichoptic motion coherence. Red dot line: the bias of the test; black dot line: 95% limits of agreement.

In order to show whether the instability between trials would result in any measurable bias, a test-retest reliability of the two binocular function tests was evaluated in another ten participants with amblyopia. Pearson’s correlation coefficient (r) and Bland-Altman difference plot^46^ are shown in Fig. 6. Linear correlation is found when ECR values of the 2^nd^ test are plot against the 1^st^ test in this group (phase: r = 0.7577, p = 0.01; motion: r = 0.7461, p = 0.02). No bias was detected by Bland-Altman difference plot while the mean difference between the 1^st^ and 2^nd^ tests was not statistically different from zero (phase: t_9_ = 1.315, p = 0.22; motion: t_7_ = 0.736, p = 0.527). However, we noted that a few dots located outside the lower and upper limits of 95%, a relative small sample size and the intervention in this group might have increased the variability our results.

### Correlation between the change of monocular and binocular function in amblyopia

**Figure 7:**
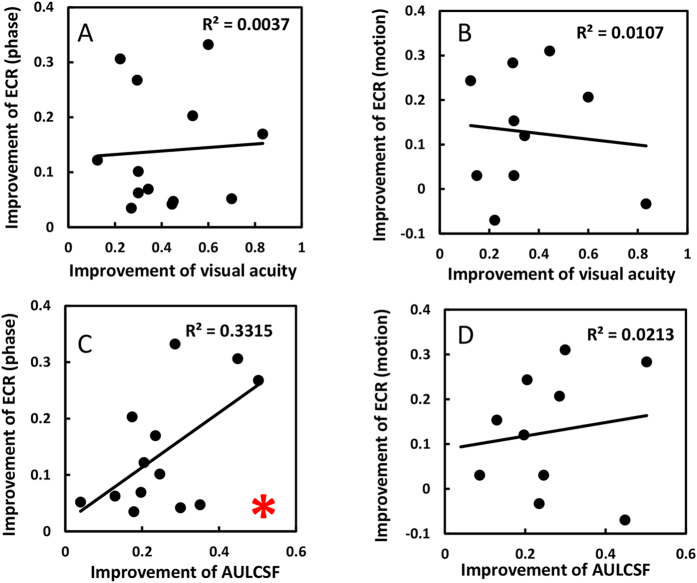
Relationship between the improvements of monocular and binocular function. (**A**) visual acuity and binocular phase combination; (**B**) visual acuity and dichoptic motion coherence; (**C**) AULCSF and binocular phase combination and (**D**) AULCSF and dichoptic motion coherence. The asterisk indicates correlation of statistical significance.

It is suggested that binocular combination of supra-threshold stimuli does not merely depend on the interocular difference in contrast sensitivities^37^,^38^ because of the inhibitory interaction from the non-amblyopic eye. It is shown to be the case here. Neither interocular difference of AULCSFs before nor after training made a good predictor of ECRs in phase and motion tasks (all p>0.50). Consistent with what was found recently^25^, we didn’t found any correlation between the VA and binocular combination improvements (Fig. 7A,B: VA vs phase: Pearson’s r = 0.061, p = 0.843; VA vs motion: Pearson’s r = 0.103, p = 0.777). However, it is interesting to find that the improvement of AULCSF correlates with the improvements of ECR in phase combination (Fig. 7C, Pearson’s r = 0.576, p = 0.039), which suggests that when AULCSF is used as an index, the relationship between monocular and binocular function improvement could be revealed. For the motion coherence task (Fig. 7D), which may reflect the function of dorsal stream, no correlation is found (Pearson’s r = 0.146, p = 0.688).

### CSFs and binocular phase combination in normal participants with ND filter

It is clear that modulation of mean luminance with ND filters in one eye results in abnormal binocular combination in normal participants^47^. In order to further elicit how the change of AULCSF contributed to the change of ECR phase combination, we have applied different densities of ND filters to three normal participants to simulate “amblyopic” vision. For simplicity, we did not calculate the gain-control efficiency and transducer non-linearity separately but used ECR instead to indicate the overall effect of ND filters. In order to be comparable with the data obtained from the amblyopic participants, we set the measurements with filter strength of 2.1 ND as baseline.

**Figure 8:**
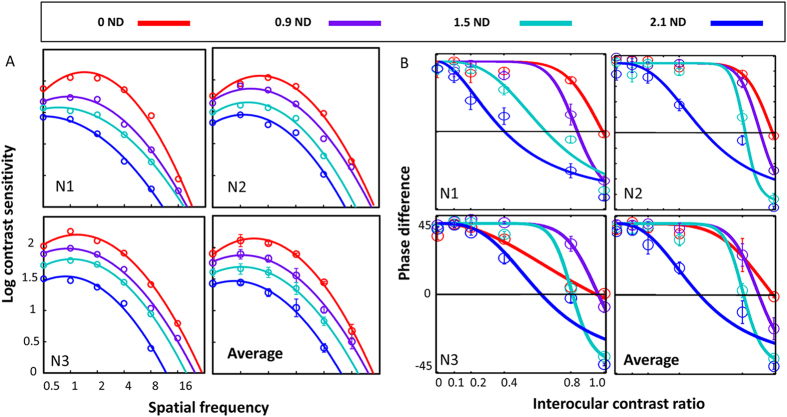
AULCSFs and ECRs of phase combination in normal participants with different density of ND filters: Red: 0 ND; purple: 0.9 ND; green: 1.5 ND; blue: 2.1 ND. (**A**) AULCSF; (**B**) ECR of phase combination. The non-activated shutter goggles were worn during measurements of CS and acted as a 0.3 unit ND filter.

Our study has replicated what was found before^47^. One-way ANOVA shows that by reducing the mean luminance in one eye, the AULCSF decreased monotonically from 9.12 to 4.82 (Fig. 8A; F_3,10_ = 29.74, p < 0.0001), and ECR of phase combination decreased from 0.97 to 0.53 (Fig. 8B; F_3,10_ = 18.4, p = 0.001). It has clearly been shown that the change in AULCSF and ECR correlated with each other (Pearson’s r = 0.83, p = 0.006). When plotted together (not shown), comparison of the regression coefficients shows the slopes of the best fitting linear functions do not differ significantly between groups (t = 0.80, p = 0.434). In other words, the change of monocular AULCSF predicts what is expected to be observed in the binocular ECR task both in amblyopic and normal participants.

### Monocular or binocular effect?

Thanks to the suggestion of an anonymous referee, we further analyzed whether or not the improvement of binocular function was due to a pure monocular mechanism. It is hypothesized that monocular attenuation affects the binocular summation through both the signal path and the gain-control path, in which the total contrast energy (TCE) played an important role in the asymmetric interocular suppression in amblyopia. TCE is jointly determined by three factors^40^,^48^: (1) the apparent contrast of the input; (2) the gain-control threshold at which contrast gain control become apparent; (3) the transducer non-linearity. Thus, the ECR calculated by Huang’s model in fact reflects both the effect of monocular attenuation and interocular suppression.

Three participants with anisometropic amblyopia took part in this experiment. Details are listed in Table S3. They were trained with monocular contrast detection task similar to what was described above. Direct comparisons of CSFs and phase combination before and after training were performed. Specifically, over-corrected lens were given after training in order to assess whether and how binocular combination would be affected by monocular attenuation.

**Figure 9:**
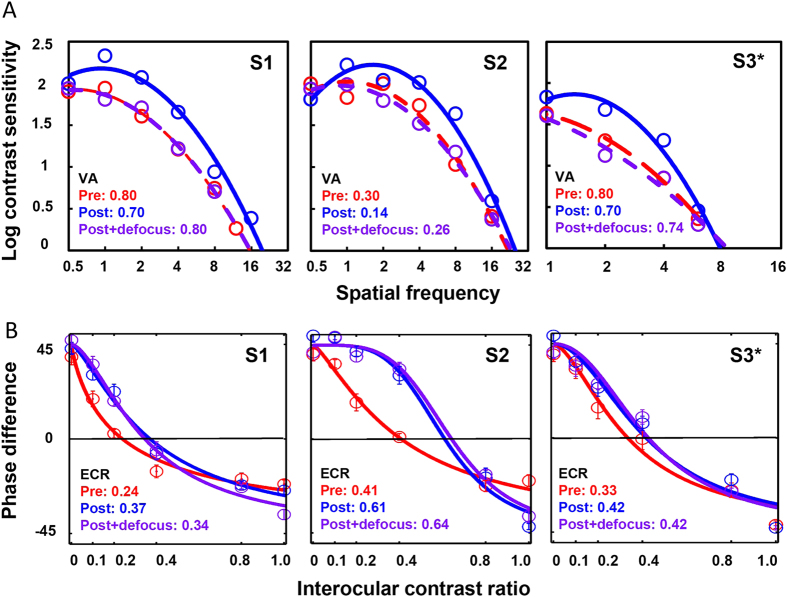
(**A**) CSFs of three participants before (red dashed line), after (blue solid line) training and after training + defocus (purple dashed line). The over-corrected lens was carefully selected so that the measurements of VA and CSF were comparable with pre-training data (details could be found in SI Table S3). (**B**) ECR of the phase combination in the same participants under the same condition. Noted that phase combination remained the same even the CSFs were degraded by optical defocus to the pre-training level. *indicated that S3 was trained and measured with tilted gratings +/–5 degree deviated from horizontal orientation instead of a pair of orthogonal ones.

If perceptual learning just changed the input contrast of the amblyopic eyes without modifying the contrast gain control thresholds, identical contrast sensitivity as pre-training measurement induced by optical defocus after training would have resulted in identical ECR. As shown in Fig. 9, training improves CSFs of the three participants. The over-corrected lens was carefully selected so that the measurements of VA and CSF were comparable with pre-training data. Surprisingly, while we found that ECRs increased after monocular training (S1: from 0.24 to 0.37; S2: from 0.41 to 0.61; S3: from 0.33 to 0.42), the improvement retained even with monocular defocus of the amblyopic eyes (S1: 0.34; S2: 0.64; S3: 0.42). The explanation is not straight-forward if only monocular effect is considered, therefore the contrast gain threshold should also be involved in order to explain why identical CSFs gave rise to different ECRs. In this case, binocular combination is changed after monocular perceptual learning, possibly due to the change of TCE by mixed mechanism and it is not solely the monocular effect.

## Discussion

In this study, we investigated whether monocular perceptual training transferred to improvements in binocular function among anisometropic amblyopes. Learning effects did not only translate to improved contrast sensitivity and VA outcomes but in this paradigm, improvements were also noted using novel psychophysical approaches to binocular function assessments. More importantly, when AULCSF and ECR phase combination were used as this index, a relationship between monocular and binocular improvement was observed.

Our perceptual learning data were in good agreement with previous studies^11^,^12^,^49^. Contrast detection using a similar paradigm in amblyopes induced about 10 dBs improvement in contrast sensitivity^12^, which was similar to the results observed here. Since our task directly focused on a specific spatial frequency, it was likely that corresponding improvements were maximized; thus, higher (about two folds) than what was reported previously in our dichoptic training study^18^. A generalization of 3.7 octaves across spatial channels was closed to the 4.04 octaves reported elsewhere, and it was much broader than the width estimated by the first-order spatial frequency channels^50^. Our participants also achieved 1.64 lines of VA improvement, which was similar to other studies using a monocular training paradigm^5^,^13^.

Several models explained binocular interaction in amblyopia to account for the common deficits observed^31^,^37^,^38^,^40^,^51^. Most agreed that amblyopes exhibited higher signal attenuation and interocular inhibition. It is reasonable to address: (1) what relationship existed between monocular performance and binocular combination and (2) whether improvements in monocular performance correlated with improvements in binocular functions. For the first question, our result was consistent with the findings that contrast sensitivity function alone could not account for the abnormal binocular interaction in amblyopia. On the second note; however, it was interesting to find that AULCSF, now regarded as a comprehensive measurement of our “visual world”^29^, served as a good metric in explaining binocular phase combination changes observed due to perceptual learning. Although a broad metric, AULCSF worked better than any single point measurements such as VA, in predicting binocular function outcomes. We felt that the total contrast energy (TCE) hypothesis may help to understand this result. As pointed out previously^39^,^40^, the binocular contrast gain control is determined by TCE, a parameter describing the overall contribution of the environment across spatial frequencies and orientations. Perceptual training using contrast detection in amblyopia improved contrast sensitivity at their trained spatial frequency as well as neighborhood spatial channels with broader bandwidth than the normal visual system. Therefore, it is possible that the improved TCE in the amblyopic eye during perceptual learning allowed for a greater potential in binocular function improvements. Notably, when we consider TCE to be a factor modulating binocular combination, mechanism of monocular inputs itself was insufficient and altered interocular interaction should also be taken into accounted.

The results from our normal participants with ND filters gave additional support to the link between AULCSF and binocular combination. It was believed that attenuation of mean luminance in one eye in normal observers can disrupt binocular combination^47^,^52^,^53^, stereopsis^54^ without changing the physical contrast of the stimuli. This effect was explained by the delay of signal transduction or the attenuated contrast gain in one eye when photon reception was reduced under low illumination. This process likely originated in the pre-cortical regions^55^. Again, we found that the improvement of AULCSF and binocular combination correlated with each other, weighting similarly in amblyopic and normal participants. Overall, 10% of AULCSF improvement contributed to around 5% of effective contrast in the amblyopic eye in binocular phase combination task.

Why monocular perceptual learning induced binocular function improvement in a similar way as the front-end modulation of retinal illumination? The similarity shown here implied that perceptual learning of contrast detection occurred at early or midlevel visual area, in line with the hypothesis that binocular summation stage remained intact in amblyopia. Bejjanki *et al.*^56^ introduced a neuronal model suggesting perceptual learning strengthened feed forward connectivity from LGN to V1. If this was the case, it may be possible that learning took place before binocular signal combination so that it was specific to the trained eye and contributed to binocular combination. To clarify, we are not arguing that enhanced binocular performance was due after monocular perceptual learning, as there was also an independent study that showed improvement of CSF did not translate directly to effective contrast in binocular combination^57^. We feel that the severity of amblyopia and the difficulty of the task were important factors determining the interpretation of the results. It was also worth noting that our patients remained far from total recovery of binocular functions (phase ECR around 50%, motion ECR around 30% after training), while dichoptic learning might lead to higher magnitude of VA improvement and interocular suppression alleviation^15^,^25^,^58^. A question remained whether further improvement in binocular function could be observed in this group of participants if they participated in additional anti-suppression therapies. A well-designed control study was needed to systematically compare the effect of monocular and dichoptic perceptual learning.

There were several limitations in our study. First, the test-retest reliability was assessed in a patching group instead of a non-intervention group because of the regulation of clinical practice. They were not well matched in terms of age and interventional periods with the PL group. Though we got comparable results with a previous study^41^, we tried to avoid any direct comparison between these two groups, thus the strength of the results was weakened. Second, it was suggested that short-term monocular deprivation may boost the contribution from the patched eye in binocular combination^59^,^60^,^61^; thus, confounding factors would have been introduced by this effect. However, it was not likely to be the cause of the lack of improvement in binocular function of the patching group for two reasons: (1) this transient effect lasted for a short time and diminished after 30 minutes of patching cessation^59^; (2) participants in the PL group also performed monocular training with their non-amblyopic eyes patched.

Our results had potential implications for clinical application. This proof-of-principle study revealed that monocular perceptual learning in contrast detection at a specific spatial frequency could be generalized to binocular combination, albeit with subtle effect size. Future study concerning binocular function of amblyopia should not ignore the influence of enhanced inputs from the amblyopic eyes due to any reason.

## Additional Information

**How to cite this article**: Chen, Z. *et al.* Monocular perceptual learning of contrast detection facilitates binocular combination in adults with anisometropic amblyopia. *Sci. Rep.* **6**, 20187; doi: 10.1038/srep20187 (2016).

## Supplementary Material

Supplementary Information

## Figures and Tables

**Figure 1:**
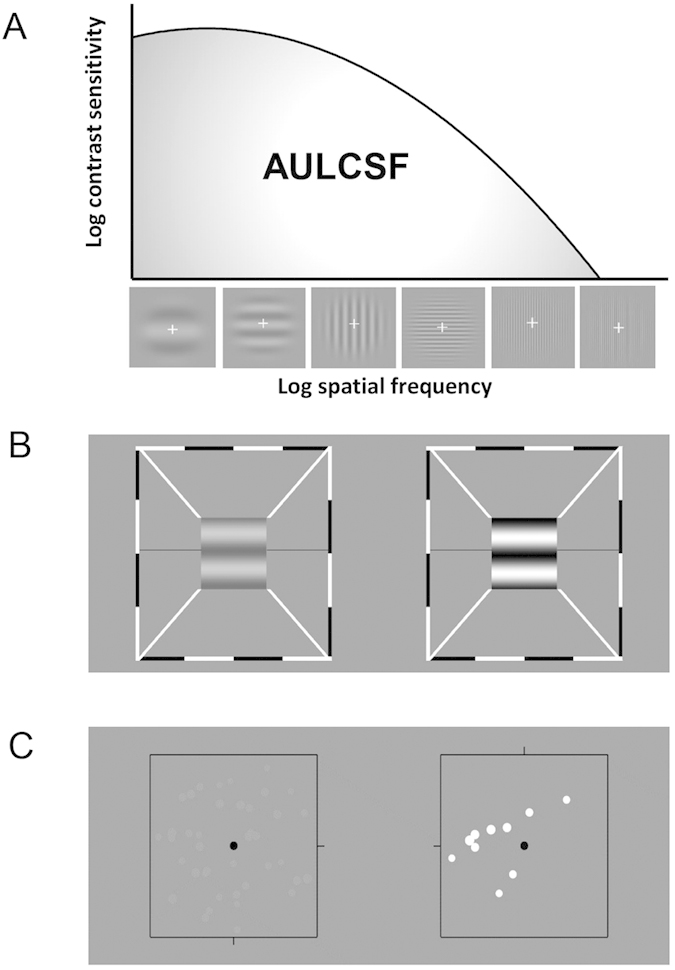
An illustration of the stimuli of psychophysical measurements. (**A**) schematic diagram of CSF and gratings used at 5 different spatial frequencies; (**B**) binocular phase combination and (**C**) dichoptic global motion coherence. The right halves in both tasks are presented to the amblyopic eyes.

**Table 1:** Baseline information of amblyopic observers.

	PL group	Patching group	p-value
IAD (logMAR)	0.48 ± 0.26	0.53 ± 0.16	0.85
Age (yrs)	18.07 ± 7.84	10.46 ± 3.08	0.01
Sex (male/female)	6/7	4/6	0.64
SED (diopter)	4.50 ± 1.79	3.73 ± 1.56	0.32
Stereo sensitivity (1/arc second)	0.0016 ± 0.003	0.0008 ± 0.001	0.41
Follow-up duration (days)	9.46	21.31	0.01

IAD: interocular visual acuity difference; SED: interocular spherical equivalence difference. Noted that baseline information in the two groups was comparable except for the age and follow-up period.
