# Supplementary figures and images for: Monocular perceptual learning of contrast detection facilitates binocular combination in adults with anisometropic amblyopia
Source: Sci Rep. 2016 Feb 1;6:20187. doi: 10.1038/srep20187 (PMC4735338; doi:10.1038/srep20187)

A

Log contrast sensitivity

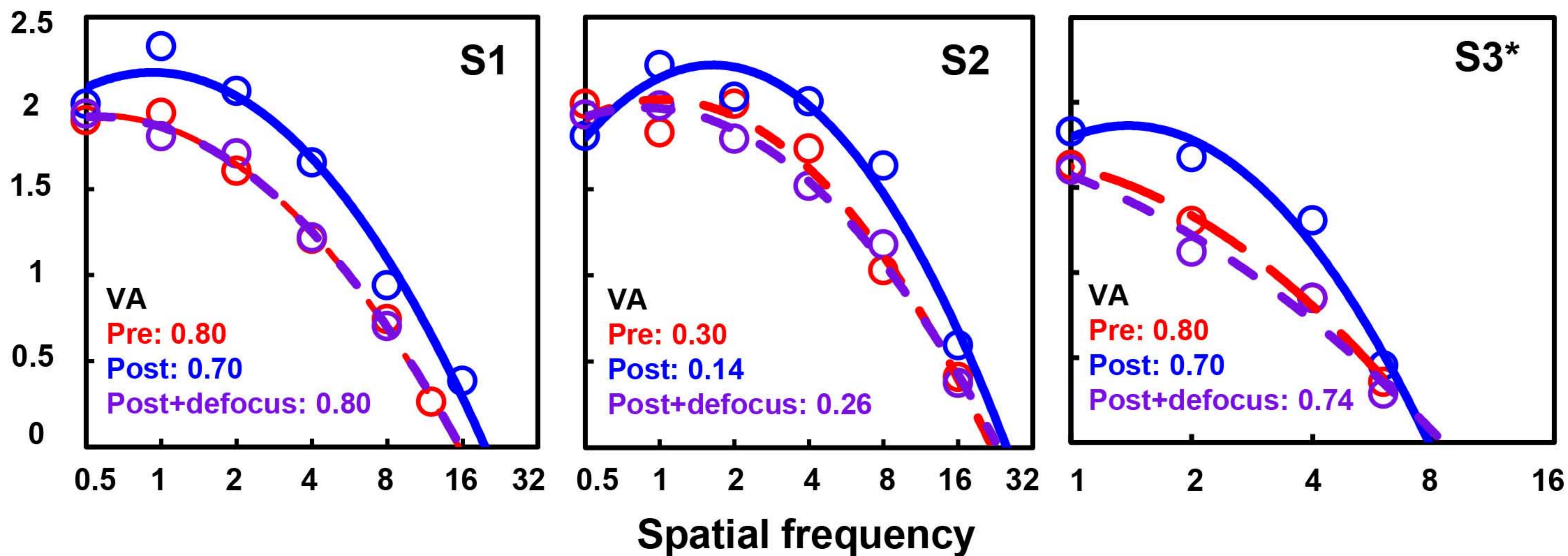

B

Phase difference

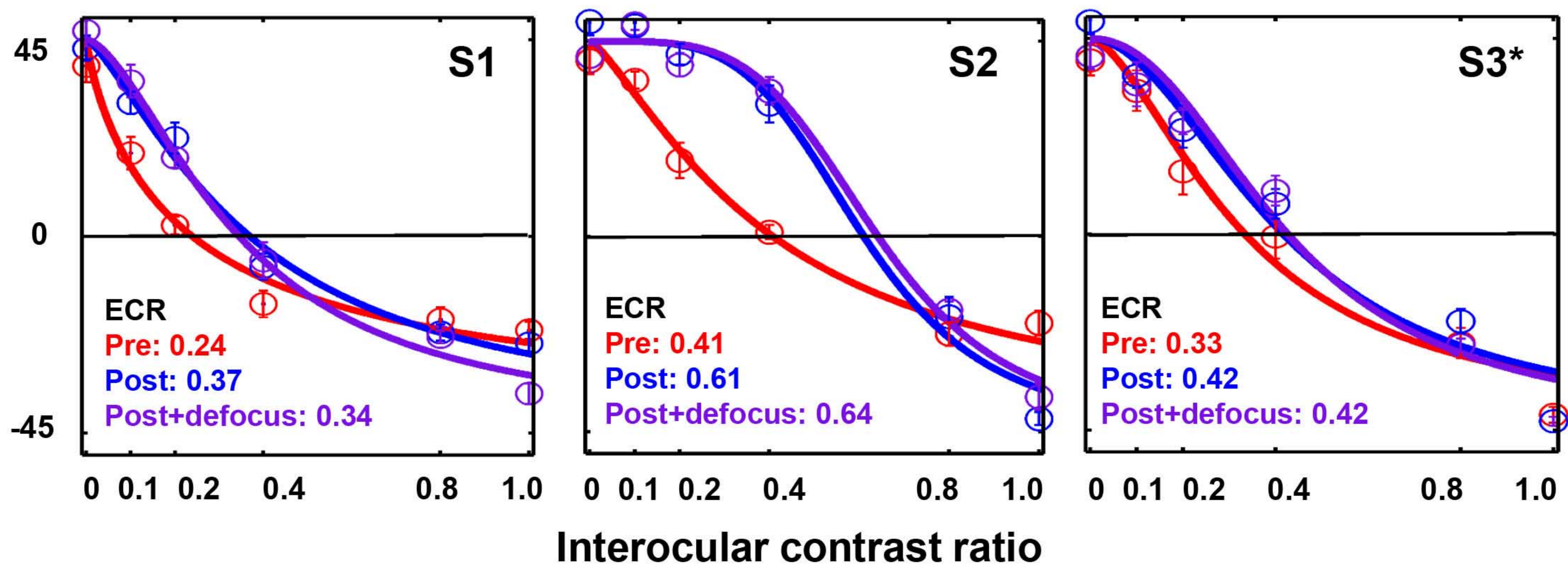

Supplement: Supplementary Information [file srep20187-s1.pdf]
